# Application of feature tracking at cine cardiac MR for the semiautomated prediction of normal right ventricular systolic function: a feasibility study

**DOI:** 10.1186/1532-429X-16-S1-P74

**Published:** 2014-01-16

**Authors:** Marcos Botelho, Jad Bou Ayache, Abraham Bogachkov, Ramin Artang, Xiaoming Bi, Monica R Vazquez, James C Carr, Jeremy D Collins

**Affiliations:** 1 Radiology, Northwestern University, Chicago, Illinois, USA; 2 Feinberg School of Medicine, Chicago, Illinois, USA; 3 Cardiovascular MR R&D, Siemens Healthcare, Los Angeles, California, USA; 4 Cardiology, University of Nebraska Medical Center, Omaha, Nebraska, USA; 5 Cardiology, La Moraleja Hospital, Madrid, Spain

## Background

Cardiac MR (CMR) has emerged as the gold standard for the evaluation of biventricular systolic function. Semi-automated algorithms for investigating left ventricular systolic function exist, but application to the right ventricle (RV) is challenging. Recently, feature tracking has been developed to semi-automatically track the tricuspid annulus on 4-chamber cine imaging. The purpose of this feasibility study is to determine the ability of semi-automated quantification of the CMR tricuspid annular plane systolic excursion (CMR-TAPSE) to predict normal RV systolic function, comparing to echocardiography determined TAPSE (echo-TAPSE) and quantitative assessment of RV systolic function at CMR.

## Methods

Retrospective analysis of 15 patients (10 males, avg age 51.7 years) referred for cardiac MR and transthoracic echocardiography with suspected infiltrative heart disease. MR images were acquired at 1.5T (MAGNETOM Avanto or Espree, Siemens AG, Healthcare Sector, Erlangen, Germany). A high temporal resolution 4-chamber segmented cine bSSFP acquisition was acquired (TR/TE 2.7/1.1 msec, FA 69 degrees, 50 phases, BW 933, parallel factor 2, temp res = 21.4 msec). Standard temporal resolution bSSFP cine short axis images were acquired for quantitative evaluation of RV systolic function (TR/TE 2.8/1.4, FA 79 degrees, 25 phases, BW 933, parallel factor 2, temp res = 39.2 msec). 4-chamber HR bSSFP cine images were analyzed using prototype software (Siemens Corporation, Corporate Technology, Princeton, New Jersey) evaluating deformation fields to semi-automatically identify and track the tricuspid base plane at the lateral tricuspid insertion determining CMR-TAPSE. The CMR RV ejection fraction (RVEF) was determined on a dedicated workstation (QMass 7.2, Medis, Leiden, Netherlands) by two reviewers. A single reviewer calculated echo-TAPSE on a dedicated PACS workstation (Synapse Cardiovascular, Fujifilm Medical Systems USA). CMR-TAPSE and Echo-TAPSE were correlated with the CMR RVEF using Pearson's correlation. ROC analysis was performed using a TAPSE threshold of 15 mm and defining normal RV systolic function as a RVEF≥40%.

## Results

**Figure 1:**
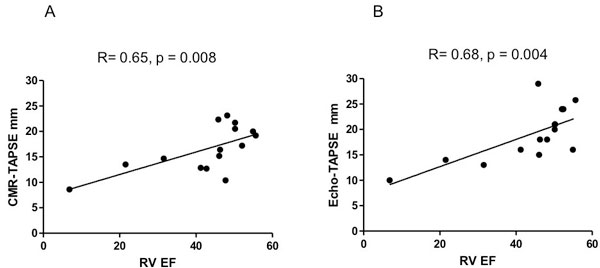
**Correlations between A) CMR-TAPSE and RVEF, B) Echo-TAPSE and RVEF**. RVEF: right ventricular ejection fraction, CMR-TAPSE: cardiac MR determined tricuspid annular plane systolic excursion, Echo-TAPSE: transthoracic echocardiographically determined tricuspid annular plane systolic excursion.

**Figure 2:**
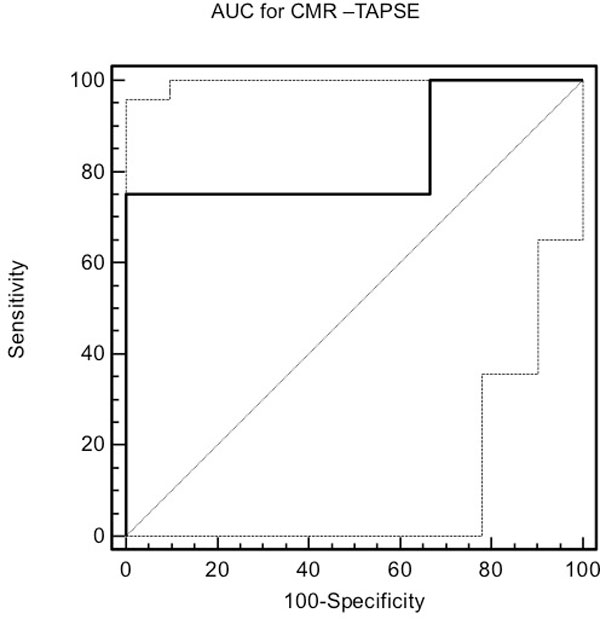
**ROC analysis for CMR-TAPSE to predict a RVEF > 40%**. The area under the ROC curve is 0.833 (95% CI 0.556-0.968, p = 0.004), with a sensitivity of 75% and a specificity of 100%.

There was a significant correlation between CMR-TAPSE and CMR RVEF (R = 0.65, p = 0.008) (Figure 1). Correlation between echo-TAPSE and CMR RVEF was comparable (R = 0.68, p = 0.004). CMR-TAPSE underestimated but correlated modestly with echo-TAPSE (R = 0.49, p = 0.065). For CMR-TAPSE, ROC analysis generated an AUC of 0.833 (95% CI 0.556-0.968) p = 0.004 (Figure 2), with sensitivity = 75% and specificity = 100%. For Echo-TAPSE, ROC analysis generated an AUC of 1.0 (95% CI 0.780-1.000) p = 0 with sensitivity = 100% and specificity = 100%.

## Conclusions

CMR-TAPSE shows promise for efficient prediction of normal RV systolic function with similar performance to Echo-TAPSE and good correlation with CMR derived RVEF. Work is ongoing to validate our results in a larger cohort.

## Funding

None.

